# Meta-q-plate for complex beam shaping

**DOI:** 10.1038/srep25528

**Published:** 2016-05-06

**Authors:** Wei Ji, Chun-Hong Lee, Peng Chen, Wei Hu, Yang Ming, Lijian Zhang, Tsung-Hsien Lin, Vladimir Chigrinov, Yan-Qing Lu

**Affiliations:** 1 National Laboratory of Solid State Microstructures, Collaborative Innovation Center of Advanced Microstructures and College of Engineering and Applied Sciences, Nanjing University, Nanjing 210093, China; 2 Department of Photonics, National Sun Yat-sen University, Kaohsiung, Taiwan 80424, R.O.C; 3 Center for Display Research, Department of Electronic and Computer Engineering, Hong Kong University of Science and Technology, Clear Water Bay, Kowloon, Hong Kong, China

## Abstract

Optical beam shaping plays a key role in optics and photonics. In this work, meta-q-plate featured by arbitrarily space-variant optical axes is proposed and demonstrated via liquid crystal photoalignment based on a polarization-sensitive alignment agent and a dynamic micro-lithography system. Meta-q-plates with multiple-, azimuthally/radially variant topological charges and initial azimuthal angles are fabricated. Accordingly, complex beams with elliptical, asymmetrical, multi-ringed and hurricane transverse profiles are generated, making the manipulation of optical vortex up to an unprecedented flexibility. The evolution, handedness and Michelson interferogram of the hurricane one are theoretically analysed and experimentally verified. The design facilitates the manipulation of polarization and spatial degrees of freedom of light in a point-to-point manner. The realization of meta-q-plate drastically enhances the capability of beam shaping and may pave a bright way towards optical manipulations, OAM based informatics, quantum optics and other fields.

Beam shaping induced by manipulation of multiple degrees of freedom of light has attracted growing attentions recently. Typical cases including Bessel beams^1^, Airy beams^2^, vector beams^3^ and vortex beams^4^, and their diverse applications in optical trapping^5^, material processing^6^,^7^,^8^, bioimaging^9^,^10^, *etc*. have been studied intensively. Generally, these specific beams can be generated via particular spatial amplitude or phase control^11^. Yet to date the available structures of the optical field are still quite limited due to the constraint of present techniques of light manipulation. Here we develop a novel design to control the polarization and spatial degrees of freedom of light in a point-to-point manner. As an example, we fabricate a device that advances the engineering of optical vortex (OV).

OV is a particular type of optical beams that carries orbital angular momentum (OAM) of photons^12^. The OAM adds a new degree of freedom in the manipulation of light, thus paving a bright way towards broadband optical communication^13^,^14^, quantum informatics^15^,^16^,^17^ and optical tweezers^18^,^19^. Among various techniques for OV generation, a device called q-plate is especially attractive due to the coupling of spin angular momentum (SAM) and OAM^20^. The q-plate not only plays an important role in classical optics^21^, but also enables the alignment-free quantum communication^22^, ultra-sensitive angular measurements^23^ and other quantum-optical applications^24^,^25^. As shown in Fig. 1, it is a half-wave plate with space-variant optical axes in the transverse plane^20^. The number *q*, which denotes the changing rate of the optical axis with respect to the azimuthal angle, depicts the specific geometry. Owing to the pronounced optical birefringence and controllable director distribution, liquid crystals (LCs) become the first choice for q-plate fabrication. The objective LC orientations can be obtained via self-assembled^26^,^27^ or extra-field induced^28^,^29^ LC topological defects, and circular rubbing^30^ or photoalignment technique^31^,^32^,^33^,^34^,^35^. Till now, the reported q-plates are restricted to azimuthally variant optical axes. If we can overcome this limit, the capability of wavefront manipulation will be drastically enhanced, thus more complex beam shaping is achievable.

In this work, we propose the concept of meta-q-plate, which is featured by arbitrarily space-variant optical axes. The meta-q-plate is demonstrated using LC whose azimuthal director is photoaligned via a polarization-sensitive alignment agent. The specific space-variant alignment is realized by a dynamic micro-lithography system through a multi-step partly-overlapping exposure^36^. By this means, we fabricate meta-q-plates with multiple-, azimuthally/radially variant *q* and initial director. Various complex beams with elliptical, asymmetrical, multi-ringed, and even hurricane transverse profiles are generated, making the manipulation of optical vortex up to an unprecedented flexibility. The beam shaping and OAM steering of meta-q-plate with radial variant *q* are theoretically analysed and experimentally verified. The meta-q-plate supplies a novel method to control the polarization and spatial degrees of freedom of light, and even has potential in tailoring optical field in all degrees of freedom.

## Results

### Design and fabrication of meta-q-plate

In a q-plate, the optical axis orientation *α* with respect to the x axis follows the equation: *α*(*r*, *φ*) = *qφ* + *α*_0_, where *r* is the polar radius, *φ* is the azimuthal angle, *q* is the topological charge, and *α*_0_ is the initial angle when *φ* = 0. When a circularly polarized light beam with OAM states of *m* traverses the q-plate, an OAM variation of ±2*q*ћ is imposed. Herein, the sign depends on the input polarization, positive for left circular polarization and negative for right circular polarization. The output polarization is sign-inverted^37^. By means of traditional fabrication methods, only single *q* and *α*_0_, and simple azimuthally changing *q* could be achieved^32^,^33^. If the *q* and *α*_0_ could be arbitrarily changed along *r* and *φ*, the capability of beam shaping would be drastically enhanced and the manipulation of optical beams in a point-to-point manner is possible. To distinguish from traditional q-plate, we call it meta-q-plate, of which the optical axis distribution is much more complicated. It can be classified into four categories: 1) array with different *q* or *α*_0_ values; 2) azimuthally variant *q* or *α*_0_; 3) radially variant *q* or *α*_0_; 4) the combination of above cases.

**Figure 2:**
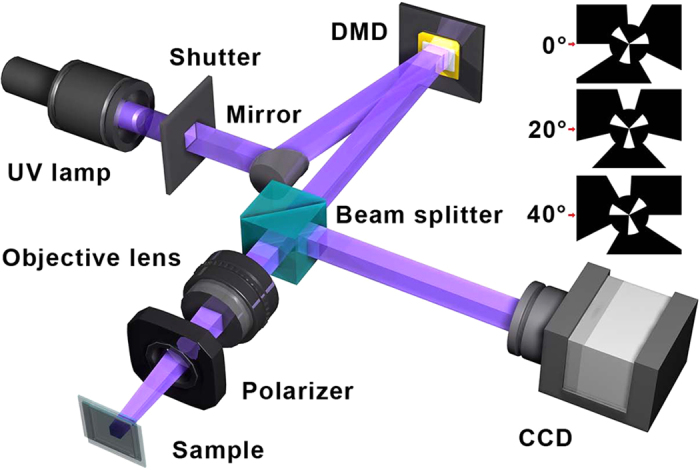
Setup for meta-q-plate fabrication. The dynamic micro-lithography setup consists of a light emission component, a dynamic pattern generation component, an image focusing component and a monitor component. A meta-q-plate with *q* = 1.5 and radially different initial angles (0 and π/2) is illustrated. Three out of all eighteen exposure sum-regions are shown as examples and corresponding polarizer angles are listed with the red arrows pointing the polar axis. CCD, charge coupled device; DMD, digital micro-mirror device.

Here, a photoalignment technique^38^,^39^,^40^ suitable for LC director control is adopted to demonstrate such a meta-q-plate. The setup is schematically illustrated in Fig. 2. A collimated UV light beam filtered at 320–500 nm (S1000, EXFO, Canada) is reflected onto the digital micro-mirror device (DMD, Discovery 3000, Texas Instruments) and subsequently carries a designed pattern. After being focused by an objective (10×, NA = 0.3, Cinv Optics Co., China), the beam passes through a polarizer, and then projects onto the alignment layers in an empty cell. The focusing process is monitored by a CCD. Here, a polarization-sensitive and rewritable sulphonic azo-dye SD1 is used as the photoalignment agent. The orientations of SD1 molecules intend to lie perpendicularly to the illuminated polarization. Besides, the SD1 is photo-rewritable and only the last polarization will be recorded after sufficient exposure. The designed *α* is recorded into the cell step by step. Firstly, the LC director distribution of certain meta-q-plate is calculated. Due to the reciprocity of LC director, the *α* modulo π is considered. Secondly, every region from 0 to π is equally divided into eighteen sub-regions, and endowed a uniform director value, from 0 to 17π/18 in intervals of π/18 respectively. Thirdly, the adjacent five sub-regions are assembled as a sum-region and exposed simultaneously. The subsequent sum-region shifts one sub-region while the polarizer rotates π/18 synchronously. Finally, after the multi-step partly-overlapping exposure^36^, each sub-region is exposed for five times with five different polarizations. Thus a quasi-continuously space-variant orientation of SD1 is accomplished. After LC E7 is infiltrated, a more continuously space-variant LC orientation is obtained due to the pronounced continuity and fluidity of LCs. Thus the designed meta-q-plate is obtained. The actual resolution is limited by the dynamic micro-lithography system and affected by the cell gap. For present system, the minimum achievable exposure region is ~1.4 μm, which is determined by both the pixel size of utilized DMD (13.68 μm × 13.68 μm) and the minification of the objective lens (10×). To accomplish the continuously space-variant orientations of SD1 and corresponding LC directors, a minimum size of ~7 μm for each exposure region should be guaranteed.

### Demonstration and characterization

**Figure 3:**
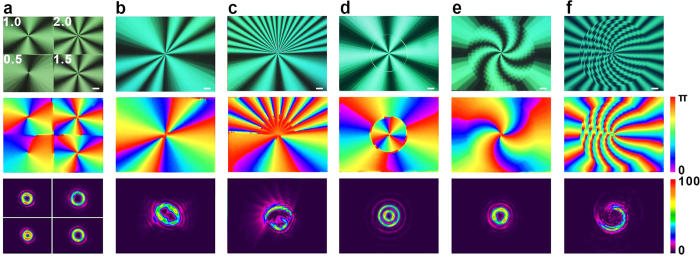
Meta-q-plates and generated complex beams. The micrographs (top), measured LC director distributions (middle) and output field patterns (bottom) of meta-q-plates: (**a**) array with q = 0.5, 1, 1.5 and 2 respectively, (**b**) azimuthally variant *q* and fixed *α*_0_ (*q* = 3 @ 0 ≤ *φ* ≤ π/2 & π ≤ *φ* ≤ 3π/2, q = 1 @ π/2 < *φ* < π & 3π/2 < *φ* < 2π, *α*_0_ = 0), (**c**) azimuthally variant *q* and fixed *α*_0_ (*q* = 10 @ 0 ≤ *φ* ≤ π, *q* = 2 @ π < *φ* < 2π, *α*_0_ = 0), (**d**) radially variant *α*_0_ and fixed *q* (*α*_0_ = 0 @ *r* ≤ 0.5r_0_, *α*_0_ = π/2 @ *r* > 0.5r_0_, *q* = 1.5) (**e**) radially variant *α*_0_ and fixed *q*, (*α*_0_ = 0 @ *r* ≤ 0.1r_0_, *α*_0_ = π/2 @ *r* > 0.9r_0_, and from the centre to the edge, *α*_0_ increases with an interval of π/18 every 0.1r_0_, *q* = 1.5), (**f**) radially variant *q* and fixed *α*_0_, (*q* = 2 @ *r* ≤ 0.1r_0_, *q* = 6.5 @ *r* > 0.9r_0_, and from the centre to the edge, *q* increases with an interval of 0.5 every 0.1r_0_, *α*_0_ = 0). All scale bars indicate 100 *μ*m. The colour bar for director distribution indicates the director varying from 0 to π, and the colour bar for output field pattern indicates the relative optical intensity.

Various meta-q-plates are demonstrated and presented in Fig. 3. Figure 3a shows a meta-q-plate which is an array with four different *q* (0.5, 1, 1.5 and 2). The micrograph gives a vivid exhibition of the sample. Under a polarized optical microscope, 4|*q*| times bright-to-dark alternation is observed. The brightness change corresponds to the variation of angles between the LC director and the polarizer. The bright domains correspond to regions with LC directors around 45° with respect to the polarizer or analyser, whereas the dark domains correspond to regions with LC directors approximately parallel to the polarizer or analyser^41^. When rotating the sample, the bright and dark regions interconvert gradually, confirming that the LC directors vary continuously and smoothly. Furthermore, the real azimuthal director distribution is examined via a two-dimensional Stokes parameters measurement. The colour variation from purple to red indicates corresponding director from 0 to π. The experimental results indicate that the design of complex optical-axis distribution has been faithfully realized, demonstrating the high precision of our method. The performance on beam shaping is also characterized. A circularly polarized 633 nm laser with a Gaussian profile scans the four parts individually and is captured by a CCD. A voltage is applied to the cell to keep half-wave retardation. Four OVs carrying different OAM according to the *q* values are generated separately. Combined with fast beam steering technique, rapid changing among different OAMs is possible^21^,^42^. This kind of meta-q-plate is suitable for the design of broad topological charge array, furthermore the design could be realized via the combination of the high quality photoalignment and dynamic photolightography with excellent image-output flexibility.

Figure 3b,c are two meta-q-plates with azimuthally variant *q* and fixed *α*_0_. Figure 3b is a sample with *q* = 3 in first and third quadrants and *q* = 1 in second and fourth quadrants. Figure 3c is a sample with *q* = 10 in the upper half and *q* = 2 in the lower half. Our technique ensures the reliable fabrication of the designed meta-q-plates. For the two-dimensional Stokes parameters measurement setup, the resolution is ~10 μm, where only one director orientation can be detected and then indicated in an individual color. If more orientations exist in a region smaller than 10 μm, details cannot be revealed. For the continuously variant director, only regions larger than ~100 μm could be clearly depicted. Therefore, the drastically changed LC orientations of upper center region in Fig. 3c cannot be accurately presented. Fortunately, the smooth LC orientation could be proved by the continuous brightness variation in corresponding micrograph. As expected, the elliptical and asymmetrical output beam profiles are obtained. Figure 3d,e are the meta-q-plates with radially variant *α*_0_ and fixed *q* of 1.5. Figure 3d is a sample with a π/2 shift of *α*_0_ at 0.5r_0_ (r_0_ is the shortest length between the centre and the edge of the exposure region). A circle is observed in the micrograph which is due to the disclination caused by director discontinuities. Correspondingly, a two-ringed OV with both topological charge and radial index is generated^43^, which may find applications in gravitational wave detection^44^ and the trapping of cold atoms^45^. Figure 3e is a sample with *α*_0_ changing from 0 to π/2 with the step of π/18 varying from the centre to the edge. The initial angle introduces an overall phase shift thus does not influence the output OAM^20^. Here, the phases at different radii are shifted differently. However, the final optical field pattern is still a single-ringed OV. Figure 3f is a sample with radially variant *q* and fixed *α*_0_ = 0. From the centre to the edge, *q* increases from 2 to 6.5 with an interval of 0.5. An optical field with a hurricane profile is observed. Theoretically, owing to the non-uniform intensity distribution and the rotational Poynting vector of such beam, the optical force may supply a powerful optical tweezer for complicated micromanipulations. As shown in these examples, our technique allows the flexible control of azimuthal optical axis at each point independently, thus could provide an arbitrary manipulation of the wavefront of the incident beam.

### Meta-q-plate with radially variant *q*

**Figure 4:**
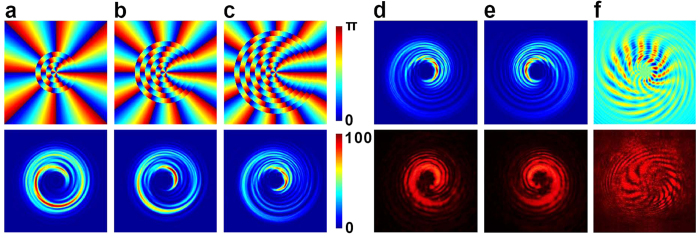
Simulation and experimental verification. Simulated LC director distributions (top) and corresponding output transverse profiles (bottom) of meta-q-plates with: (**a**) *n* = 5, *q* = 2~4, (**b**) *n* = 7, *q* = 2~5, (**c**) *n* = 9, *q* = 2~6. The colour bar for the director distribution indicates the director varying from 0 to π, and the colour bar for the transverse profile indicates the relative optical intensity. The simulated transverse profiles (top) and the corresponding experimental results (bottom) of the meta-q-plate with *n* = 10, *q* = 2~6.5 under the incident polarization of (**d**) left and (**e**) right circular handedness. (**f**) Simulated (top) and experimental (bottom) Michelson interferograms obtained by the output beam with a spherical linearly polarized reference wave.

We simulate the meta-q-plate shown in Fig. 3f to find out the cause of the special beam shape. A circularly polarized Gaussian beam propagates through the centre of the meta-q-plate and the diffracted pattern is projected on a screen. A spherical lens is inserted before the sample to produce a spherical wavefront. For our meta-q-plates, their effect on incident spherical wave can also be calculated through the Jones matrix formalism and the Fresnel diffraction integral^46^,^47^,^48^. To analyse the evolution of transverse profile from doughnut-like to hurricane shape, calculations of various meta-q-plates with radially increasing quantity (*n* = 2~10) and value (2~1.5 + 0.5*n*) of *q* are carried out. Three examples are presented in Fig. 4a–c. Since the phase front and transverse profile are dependent on *q*, here the multi-*q* introduces interference to the output optical field. The handedness of helical phase causes spatially asymmetric energy distribution. Therefore, as compared to the doughnut profile generated by traditional q-plate, the transverse profiles here gradually turn to hurricane shape. As expected, the radius of final optical field enlarges due to adding larger *q* regions. Along with the quantity of OV modes increasing, the total optical field energy decreases. In our simulation, all the optical intensities are normalized respectively for a better exhibition.

For results above, the incident polarization is left-handed. To study the handedness relationship between incident polarization and the hurricane optical profile, the cases of left and right circularly polarized incident light are simulated respectively. As shown in Fig. 4d,e, when the incident polarization changes, the handedness of the hurricane optical profiles reverses accordingly. The corresponding experimental results are in agreement with the simulations. To further characterize the wavefront generated by the meta-q-plate (*n* = 10, *q* = 2~6.5), the Michelson interferogram between the output beam and a spherical reference wave is simulated, and experimentally verified^33^. As revealed in Fig. 4f, compared to the hurricane optical field given in Fig. 4d, many radial fringes are observed. Indeed, in a q-plate with single topological charge, 2|*q*| spiral fringes are equally distributed. Thereby, our results confirm that the meta-q-plate really induces a helical wavefront. Furthermore, the density of fringes changes with azimuthal angle, indicating that a mixed *q* is carried by the beam, which may facilitate the multiplexing and demultiplexing of OAM.

## Discussion

Here we develop a novel design, namely meta-q-plate, to control the polarization and spatial degrees of freedom of light in a point-to-point manner. A technique suitable for high-quality and flexible realization of the design was developed via the combination of a polarization-sensitive alignment agent and a dynamic microlithography system. By this means, various categories of meta-q-plates were demonstrated through a multi-step and partly-overlapping exposure strategy. Meta-q-plates with multiple-, azimuthally/radially variant *q* and *α*_0_ were utilized to generate complex beams with elliptical, asymmetrical, multi-ringed and hurricane transverse profiles. Most of them are demonstrated for the first time. A hurricane optical profile generated by a meta-q-plate with radial variant *q* was theoretically analysed and experimentally verified.

The design of meta-q-plate permits an arbitrarily space-variant control of LC director, while the proposed technique can realize the design in high quality. Actually, the design could also be realized by other techniques, such as direct laser writing^34^,^35^. And the material for meta-q-plate is not limited to LCs. The specific design can also be realized in artificial birefringent materials^49^,^50^. Nevertheless, since LC cells can be advantageously used as versatile wavelength tunable phase retardation plates^51^, the proposed LC meta-q-plates eliminate the cost of preparing different elements for different wavelengths. And the phase retardation can be precisely modified through adjusting cell gap or LC birefringence, thus the absorptive electrodes are unnecessary, making the meta-q-plate suitable for broadband (visible, infrared to terahertz^52^,^53^) and intense-light applications^54^. Thanks to the rewritability of SD1, the LC orientation can be arbitrarily reconfigured^55^, enabling dynamic beam shaping. Furthermore, dynamic modulation of beam profile can also be accomplished due to the electro-optical tunability of the LCs. Vector beams can be generated as well by changing the incident polarization. Our technique drastically enhances the capability of optical beam shaping and settles a fundamental requirement in the fields of optical manipulations, micro-fabrications, OAM based informatics and quantum optics.

## Methods

### Chemicals and reagents

Indium-Tin-Oxide (ITO) coated glass substrates are ultrasonic bathed, UV-Ozone cleaned and then spin-coated with 0.5% solution of sulphonic azo-dye SD1 (Dai-Nippon Ink and Chemicals, Japan) in dimethylformamide (DMF). All cells are infiltrated with LC mixture E7.

### Cell assembling and photoaligning

Spurt 6 *μ*m spacers over one substrate then put the counter substrate over it. The two substrates are assembled together and sealed by epoxy glue. Afterwards the cell is placed at the image plane of the DMD based micro-lithography system to record the designed patterns. Each area is exposed with a dose of ca. 1 J/cm^2^ each time, and after the eighteen-step five-time-partly-overlapping exposure with a total exposure dose of 5 J/cm^2^, a quasi-continuous space-variant orientation of SD1 is carried out. After LC capillarily filled, the desired meta-q-plate is achieved.

### Characterizations

The setup for two-dimensional Stokes parameters measurement consists of a polarizer, a quarter-waveplate, a holder for samples, another quarter-waveplate and a polarizer mounted on motorized rotators in sequence (see supplementary Fig. S1). A CCD is used as a two-dimensional detector array for the simultaneous detection of all four Stokes parameters of the output optical image. A LabVIEW program is used to control the two rotators, as well as to record and calculate the data.

A Michelson interferometer is used to characterize the wavefront generated by meta-q-plate (see supplementary Fig. S2). A 633 nm linearly polarized Gaussian beam pass through a polarizer and a spherical lens (f = 100 mm) and then is equally split by a beam-splitter. One propagates through a quarter-waveplate to get a circularly polarized light. Then the beam incidents to the meta-q-plate and then interferes with the other reference beam. The interferogram is recorded on the screen and captured by a camera.

## Additional Information

**How to cite this article**: Ji, W. *et al*. Meta-q-plate for complex beam shaping. *Sci. Rep*. **6**, 25528; doi: 10.1038/srep25528 (2016).

## Supplementary Material

Supplementary Information

## Figures and Tables

**Figure 1:**
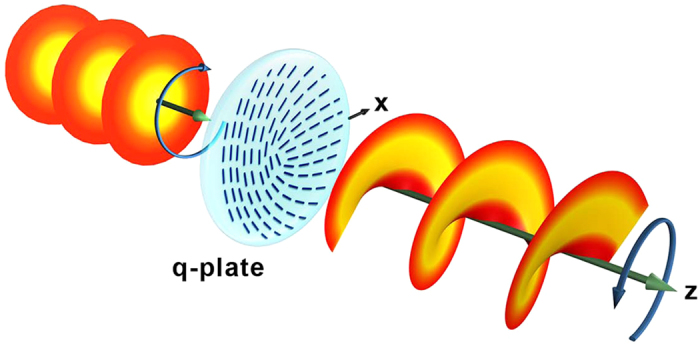
Q-plate and OV generation. Pictorial illustration of the optical action of a q-plate with *q* = 0.5 on an input left circularly polarized plane wave. The output beam is a right circularly polarized helical mode with OAM given by *m* = 1. The dark blue sticks on the q-plate depict the local LC directors. The circular arrows denote the polarization handedness from the point of view of the source. z indicates the light propagation direction and x is the polar axis.
