# Supplementary material for: Meta-q-plate for complex beam shaping
Source: Sci Rep. 2016 May 6;6:25528. doi: 10.1038/srep25528 (PMC4858674; doi:10.1038/srep25528)
Supplement: Supplementary Information [file srep25528-s1.doc]

*Supplementary Information*

**Meta-q-plate for complex beam shaping**

Wei Ji^1,+^, Chun-Hong Lee^2,+^, Peng Chen^1^, Wei Hu^1,*^, Yang Ming^1^, Lijian Zhang^1^, Tsung-Hsien Lin^2,*^, Vladimir Chigrinov^3^, and Yan-Qing Lu^1,*^

^1^ National Laboratory of Solid State Microstructures, Collaborative Innovation Center of Advanced Microstructures and College of Engineering and Applied Sciences, Nanjing University, Nanjing 210093, China.

^2^ Department of Photonics, National Sun Yat-sen University, Kaohsiung, Taiwan 80424, R.O.C.

^3^ Center for Display Research, Department of Electronic and Computer Engineering, Hong Kong University of Science and Technology, Clear Water Bay, Kowloon, Hong Kong, China.

^*^Correspondence and requests for materials should be addressed to W. H. (email: [huwei@nju.edu.cn](mailto:huwei@nju.edu.cn)), T. L. (email: [jameslin@faculty.nsysu.edu.tw](mailto:jameslin@faculty.nsysu.edu.tw)) and Y. L. (email: [yqlu@nju.edu.cn](mailto:yqlu@nju.edu.cn)).

^+^ These authors contributed equally to this work.


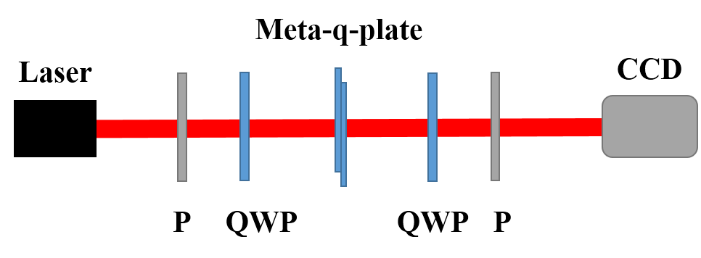


**Supplementary Figure S1. Experimental set-up for the two-dimensional Stokes parameters measurement system**. P: polarizer; QWP: quarter-waveplate; CCD: charge coupled device.


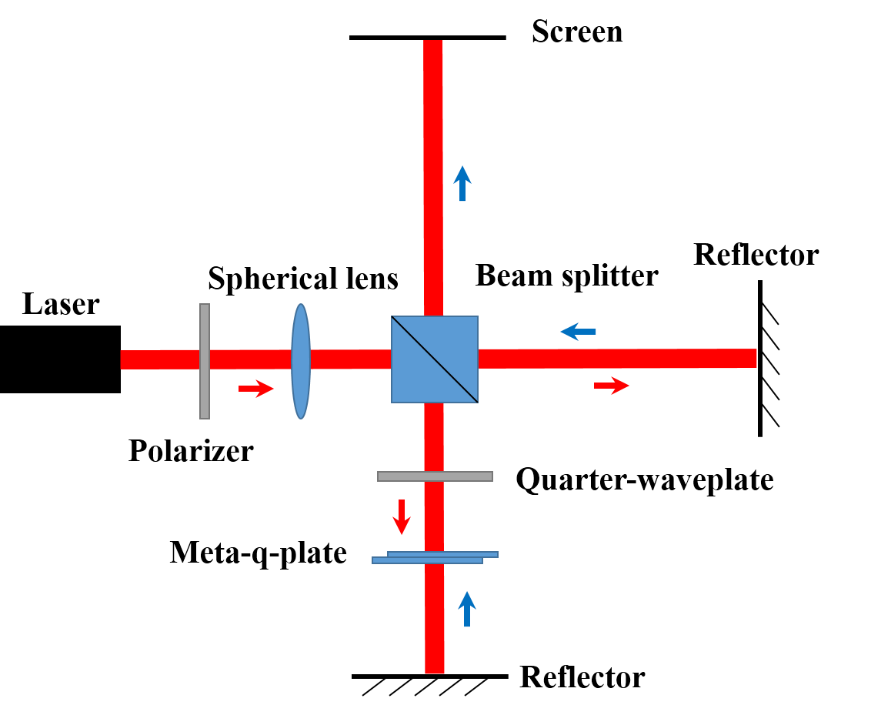


**Supplementary Figure S2. Experimental set-up for the Michelson interferometer^1^.**

A polarized Gaussian beam at 633 nm through a spherical lens was separated into two beams, namely the signal and the reference beams, by a beam splitter. The signal beam passed through a quarter-waveplate and a meta-q-plate under certain applied voltage. It was then reflected by a reflector. Notably, the signal beam passed through the meta-q-plate twice, that means the signal beam phase was also modulated twice. Since meta-q-plates function as a half-waveplate, in this case, the modulation of the phase retardation of the meta-q-plate was set to π/2 by applying appropriate voltages because the double optical path. Therefore, the reflected signal beam after passing through the meta-q-plate and the quarter-waveplate was modulated as a linearly polarized beam. The reference beam passing through the beam splitter was reflected by the other reflector and then interfered with the modulated signal beam and the interferogram was recorded on the screen and captured by a camera.

**Supplementary Reference**

1 Huang, Y. H., Li, M. S., Ko, S. W. & Fuh, A. Y. G. Helical wavefront and beam shape modulated by advanced liquid crystal q-plate fabricated via photoalignment and analyzed by Michelson’s interference. *Appl. Opt.* **52**, 6557 (2013).
